# Implant‐abutment emergence angle and profile in relation to peri‐implantitis: A systematic review

**DOI:** 10.1002/cre2.594

**Published:** 2022-06-17

**Authors:** Sara Soulami, Dagmar E. Slot, Fridus van der Weijden

**Affiliations:** ^1^ Master of Science Program of Dental Sciences Academic Centre for Dentistry Amsterdam (ACTA) University of Amsterdam and Vrije Universiteit Amsterdam Amsterdam The Netherlands; ^2^ Department of Periodontology Academic Centre for Dentistry Amsterdam (ACTA) University of Amsterdam and Vrije Universiteit Amsterdam Amsterdam The Netherlands

**Keywords:** implant‐abutment emergence angle, implant‐abutment emergence profile, peri‐implantitis

## Abstract

**Statement:**

The aim of this systematic review is to analyze literature regarding the relationship between the implant‐abutment emergence angle (EA) and implant emergence profile (EP) and the prevalence of peri‐implantitis.

**Methods:**

PubMed and the Cochrane Library were searched for studies from initiation up to April 2022. Studies describing the EA and EP in association with peri‐implantitis were considered eligible for this review and selected for inclusion in this review if implant groups with wide and narrow EA and different EP types were described.

**Results:**

Searches in PubMed and the Cochrane Library led to 1116 unique titles and the inclusion of three studies. These concerned 168–349 implants. Two studies presented the mean prevalence of peri‐implantitis which was 16.7% and 24.8% at the implant level. Both studies showed a significant relationship between peri‐implantitis in bone‐level implant groups with an EA above 30° compared to implants with an EA below 30°. A third study presented marginal bone loss which tended to be smaller when the EA was around 20°–40°. In one of the three included studies, the prevalence of peri‐implantitis was significantly higher if implants had a convex EP compared to a concave or straight EP. Another study showed a significantly higher prevalence of peri‐implantitis in implants with a convex EP compared to other EP types, if combined with an EA above 30°.

**Conclusions:**

Three eligible studies were found. Reported associations should therefore be considered with caution. Synthesis suggests an association between a larger EA (>30°) and a higher prevalence of peri‐implantitis or marginal bone loss compared to a smaller EA (<30°). A convex EP may also be associated with a higher prevalence of peri‐implantitis. However, causality remains a question.

## 1 INTRODUCTION

Peri‐implant mucositis and peri‐implantitis (peri‐implant diseases) are considered to have multifactorial etiological components that make both diagnosis and treatment difficult to approach (Dixon & London, 2019). The complex interaction of these component causes should not be ignored when associating risk factors with peri‐implant diseases (Koutouzis, 2019). Peri‐implant mucositis is defined as an inflammatory lesion of the surrounding mucosa of an implant. Peri‐mucositis has similarities to gingivitis because it is considered a reversible inflammation of the peri‐implant mucosa. Peri‐implantitis on the other hand is a nonreversible inflammation that affects mucosa and bone tissue around the implant creating loss of osseointegration (Dixon & London, 2019; Koutouzis, 2019; Lindhe & Meyle, 2008). The mean prevalence of peri‐implant mucositis in subjects with implants is estimated to be 43% while the mean prevalence of peri‐implantitis has been reported to be 22% (Figuero et al., 2014; Jepsen et al., 2015). The prevalence of replacing missing teeth with dental implants keeps increasing and is predicted to increase up to 23% by 2026 in the US population (Cochran & Froum, 2013). This creates relevance for understanding risk factors that may be associated with peri‐implant diseases. Implant design‐related factors have been identified as contributing risk factors to peri‐implant bone loss (Bain, 2003; Koutouzis, 2019; Spray et al., 2000). If the emergence profile (EP) of the restoration is excessive in such a way that the biologic width is violated, a (local) tissue reaction of the mucosa could occur, for example, redness, irritation, and edema (Starr, 1991). Bacterial colonization on implant surfaces resulting in inflammation of peri‐implant tissue has been studied (Armitage & Xenoudi, 2016; Berglundh et al., 1992; Dixon & London, 2019; Elani et al., 2018; Ericsson et al., 1992; Jepsen et al., 2015; Koutouzis, 2019; Pjetursson et al., 2012; Rinke et al., 2011; Romanos et al., 2014; Serino & Ström, 2009). The prosthetic design has also been associated with peri‐implant diseases if the prosthesis obstructs the patient in daily hygiene measures or the dental professional gaining access to surrounding surfaces for examination. It is therefore essential for long‐term peri‐implant health that an implant prosthetic design allows for efficient oral hygiene and accurate assessment to ensure early diagnosis and treatment of peri‐implantitis (Dixon & London, 2019). To accomplish this, a well‐considered and designed EP for the implant prosthesis is important (Dixon & London, 2019). The emergence angle (EA) of the implant‐abutment (angle of the implant‐abutment surface to the long axis of the implant) may be of influence the restorative design and EP (Dixon & London, 2019). The aim of this paper was to systematically review the literature concerning these two prosthetic design aspects in relation to peri‐implantitis.

## 2 MATERIALS AND METHODS

### 2.1 Research approval

The protocol of this systematic review has been submitted to the ethical committee of the Academic Center for Dentistry Amsterdam under number 2020168 and has been approved (Supporting Information: Appendix S1).

The PICO components of this review were as follows:

P = Patients with dental implants.

I = Dental implants with a large EA of the abutment and/or a convex EP.

C = Dental implants with a small EA of the abutment and/or a concave or straight profile.

O = Peri‐implant disease.

### 2.2 Focused question

What is the effect of the EA (of the implant‐abutment) and the EP (of the implant restoration) on the prevalence of peri‐implantitis in systemically healthy adult patients (≥18 years) with dental implants?

### 2.3 Search strategy

The main internet source used for this study were PubMed and the Cochrane Library. These databases were searched from initiation up to April 2022. Three search strategies were used in PubMed and the Cochrane Library to include any published study that addressed the effect of EA of implant abutments and EP of implant‐supported restorations on peri‐implant disease or peri‐implant health. Choice of keywords and controlled vocabulary were aimed to find as much relevant literature as possible concerning the prosthetic design aspects of implant restoration and its relation to peri‐implant disease. Search terms used for PubMed and Cochrane were customized according to the database being searched. The used search terms and strategies can be found in Table 1.

**Table 1:** Keywords and search strategy in PubMed and the Cochrane Library

Keywords and search strategy in PubMed
The following strategy was used in searches:
(<intervention AND outcome>)
1. Abutment AND (dental implants [MeSH Terms] OR dental implants, single‐tooth [MeSH Terms] OR dental implant* OR abutment design OR abutment OR dental prosthesis [MeSH Terms]) AND (peri‐implant health OR biologic width OR peri‐implantitis OR peri‐implantitis [MeSH Terms]) 2. Dental implants [MeSH Terms] OR dental implants, single‐tooth [MeSH Terms] OR dental implant* OR abutment design OR abutment OR dental prosthesis [MeSH Terms]) AND (peri‐implant health OR biologic width OR peri‐implantitis OR peri‐implantitis [MeSH Terms]) AND (emergence profile OR restoration profile OR concave OR convex OR straight) 3. (Emergence angle implant)
Keywords and search strategy in the Cochrane library
The following strategy was used in searches:
(<intervention AND outcome>)
1. (Dental Abutments [MeSH Terms] AND Dental Implants, Single‐tooth [MeSH Terms] OR Dental Implant‐Abutment Design [MeSH Terms] AND Peri‐Implantitis [MeSH Terms]) 2. (Dental Abutments [MeSH terms] AND Dental Implants, Single‐Tooth [MeSH terms] OR Dental Implant‐Abutment Design [MeSH terms] OR Dental Prosthesis [MeSH terms] AND emergence profile OR Biologic width and peri‐implantitis 3. (Emergence angle implant)

*Note*: The asterisk (*) was used as a truncation symbol.

### 2.4 Screening and selection

Preferably, only papers written in the English language were included to be evaluated. Articles were excluded if titles did not comply with selection criteria. Furthermore, studies were included for full‐text reading based on variables of interest. Screening for eligible articles was independently performed by two reviewers (Sara Soulami and Fridus van der Weijden). Initial screening was based on title and abstract. Papers were selected for full‐text reading if keywords based on variables of interest were present in the title and abstract. If the two reviewers disagreed, a third reviewer (Dagmar E. Slot) was conclusive in the final decision. The reference lists of the included studies were hand‐searched to identify additional studies that might potentially be relevant.

### 2.5 Selection criteria

Studies to qualify for this systematic review described the effect of dental implant‐abutment EA and implant restoration EP on peri‐implant health.

Study designs considered eligible were as follows:

– (randomized) controlled clinical trials,
– cohort studies,
– cross‐sectional studies, and
– case‐control studies.

Inclusion criteria were as follows:

– conducted in human subjects,
– adult patients (≥ 18 yrs.),
– systemically healthy patients, and
– dental implants.

Implant restoration prosthetic design aspects of interest were as follows:

– EA (of implants placed next to adjacent teeth) and
– EP.

Outcome variables of interest were as follows:

– peri‐implant disease (peri‐implantitis, perimucositis) and
– peri‐implant health.

Exclusion criteria were as follows:

– narrative reviews and case reports,
– abutment angles other than the EA, and
– implant restoration with (partial) dentures.

### 2.6 Assessment of heterogeneity

Factors to assess heterogeneity of the selected studies were based on differences in the study design aspect, risk of bias, (clinical) subject characteristics, clinical interventions, clinical outcomes, and confounding factors control.

### 2.7 Data extraction and analysis

Studies that were selected based on the focus of the research question—including details of the population, intervention, comparison outcome, and study characteristics—were processed for data extraction. If available, baseline, end, and incremental data, and means and standard deviations (SDs) were extracted by two reviewers acting independently (Sara Soulami and Fridus van der Weijden). Differences of opinion were discussed. In case of disagreement, the opinion of the third reviewer (Dagmar E. Slot) was decisive. The corresponding authors were sent email letters requesting additional information or clarification of data. Data were assessed and summarized based on study design, participants, sample size, and outcome variables. If the presented *p*‐values of outcomes in the included papers were below .05, the effects were considered statistically significant. If outcomes of the included studies were homogenous, a meta‐analysis would be performed and statistical heterogeneity would be analyzed. Otherwise, only a weighted mean of the study outcomes could be calculated.

### 2.8 Risk of bias

To assess the risk of bias across studies the modified tool of the Newcastle‐Ottawa scale for cohort studies, case‐control studies, and cross‐sectional studies was used (Supporting Information: Appendix S2) (Modesti et al., 2016; Wells et al., 2019; Zeng et al., 2015). If randomized‐controlled trials (RCTs) were found, the Cochrane Collaboration's tool was the choice of preference to determine the risk of bias (Zeng et al., 2015).

### 2.9 Quality of the evidence of the systematic review

The grade of recommendation, assessment, development, and evaluation (GRADE) system was used to rate the body of evidence of the selected studies in this review (Schünemann et al., 2013). Two reviewers (Sara Soulami and Fridus van der Weijden) rated the quality of evidence of eligible studies and the strength of the recommendations based on study design, risk of bias, consistency, and precision among outcomes, directness of results, detection of publication bias, and magnitude of the effect. Details of the GRADE system can be found in Supporting Information: Appendix S3.

## 3 RESULTS

### 3.1 Search and study selection

**Figure 1:**
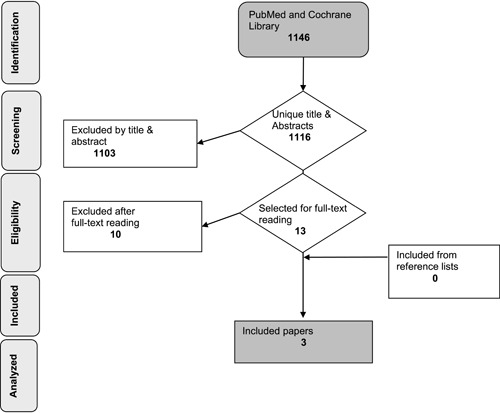
Database search and literature selection.

All three searches led to a total of 631, 96, and 229 articles, respectively, in PubMed. Three similar and modified searches in the Cochrane database resulted in 175, 7, and 8 articles, creating a combined total of 1146 (see Figure 1 (Moher et al., 2009)). A total of 1116 unique articles were found after comparing all searches and excluding identical articles. Screening of abstracts and titles resulted in 13 full‐text readings. Five of these articles were excluded because the EA and EP were not discussed. One article was a review. Furthermore, four articles were also excluded because they did not target the topic of this study and did not relate EA or EP with peri‐implant diseases (see Table 2). Subsequently, three articles were found to be eligible to address the focused question. Individual results of the selected studies are described in Table 3. We received no additional information or additional data in response to our requests.

**Table 2:** Excluded studies after full‐text reading

Reason for exclusion	Author(s) (year)
Effect of smoking on peri‐implantitis.	Galindo‐Moreno et al. (2015)
Internal versus external implant connection.	
Chipping of restoration material. Loosening of abutment screw.	Klotz et al. (2019)
Effect of metabolic diseases on peri‐implantitis.	
Preclinical study with dogs on soft tissue healing and bone	Souza et al. (2016)
Remodeling. No focus on peri‐implant disease.	
Stress distribution of bone around implants with angulated or straight abutments instead of emergence angle.	Kumar et al. (2013)
Narrative review without focus on emergence angle.	Koutouzis (2019)
Narrative review without focus on emergence angle.	Romanos et al. (2019)
Split mouth clinical trial comparing internal and external connection (no emergence angle measurements).	Pozzi et al. (2012)
Narrative review discussing emergence profile and implant‐abutment connection, no focus on emergence angle.	Dixon and London (2019)
The narrative review, addresses the emergence angle in relation to soft tissue stability and convexity of restoration, focusing on the emergence angle in relation to implant position only.	Scutellà et al. (2015)
Narrative review, discussing design features of the implant‐prosthesis‐abutment complex	Mattheos et al. (2021)

**Table 3:** Overview of study design, patient characteristics, outcome parameters, and original conclusions of the selected studies

**First author, year, title**	**Study design, duration, groups**	**Participants, implants, gender, age (mean/range), peri‐implant disease measurement, emergence angle (EA) measurement**	**Prevalence of peri‐implantitis**	**Conclusions of the original authors**
Katafuchi et al. (2018) Restoration contour is a risk indicator for peri‐implantitis: A cross‐sectional radiographic Analysis	Case‐con trol cross‐sectional study (retrospective) 1998–2003 Tissue‐level group (67) Bone‐level group (101)	96 patients, 225 implants* *57 excluded implants before radiographic analysis = 168 ♀:48 ♂:48 Mean age: 67, 6 Age range: 34–86 Peri‐implantitis data: Yes EA measurement: Yes	Bone‐level EA ≤ 30°: 15.1% (8 out of 53 implants) Bone‐level EA > 30°: 31.3% (15 out of 48 implants) Tissue‐level EA > 30°: 7.7% (3 out of 39 implants) Tissue‐level EA ≤ 30°: 7.1% (2 out of 28 implants) Bone‐level convex emergence profile: 28.8% (15 out of 52 implants) Bone‐level concave or straight emergence profile: 16.3% (8 out of 49 implants) Tissue‐level convex emergence profile: 5.9% (2 out of 34 implants) Tissue‐level concave or straight emergence profile: 9.1% (3 out of 33 implants)	An EA of >30° is a significant risk indicator for peri‐implantitis and a convex profile creates an additional risk for bone‐level implants, but not for tissue‐level implants.
Yi et al. (2020) Association of prosthetic features and peri‐implantitis: A cross‐ sectional study	Case control Cross‐sectional study (retrospective) 2002–2012 Bone‐level external connection group (215) Bone‐level internal connection group (112) Tissue‐level group (22)	169 patients, 349 implants ♀:88 ♂:81 Mean age: 58.9 Age range: Unknown Peri‐implantitis data: Yes EA measurement: Yes	EA < 30°: 8.5% (34 out of 400 surfaces (tissue‐level and bone‐level groups combined) EA ≥ 30°: 46.6% (*N*: 139 out of 297 surfaces (tissue‐level and bone‐level groups combined) Bone‐level external EA < 30°: 11.5% (29 out of 253 surfaces) Bone‐level external EA ≥ 30°: 55.9% (99 out of 177 surfaces Bone‐level internal EA < 30°: 3.1% (4 out of 127 surfaces) Bone‐level internal EA ≥ 30°s: 36.1% (35 out of 97 surfaces) Tissue‐level EA < 30°: 4.8% (1 out of 21 surfaces) Tissue‐level EA ≥ 30°: 21.7% (5 out of 23 surfaces) Convex emergence profile: 39.0% (127 out of 326 implants) Concave emergence profile: 6.1% (9 out of 147 implants) Straight emergence profile: 16.4% (37 out of 225 implants)	Overcontoured implant prosthesis is a critical local confounder for peri‐implantitis.
Inoue et al. (2020) Multivariate analysis of the influence of prosthodontic factors on peri‐implant bleeding index and marginal bone level in a molar site: A cross‐sectional study	Cross‐sectional study (retrospective) 2013– 2018 Observation period/suprastructure installation period: 45.8 months	140 patients, 310 implants ♀: 97 ♂: 43 Mean age: 61.8 ± 12.1 years Peri‐implantitis data: No (only bleeding scores and bone loss data are provided) EA measurement: Yes	Association EA and mean bleeding index *p* = .254 Association EA and marginal bone loss (MBL) *p* = .060	Our findings suggest that to reduce MBL from the perspective of prosthodontic factors it is preferable to use an implant with a taper joint connection positioned with an EA of 20°–40°.

### 3.2 Study design

The three eligible studies, Katafuchi et al. (2018), Yi et al. (2020), and Inoue et al. (2020), had a retrospective case‐control and cross‐sectional design, respectively. The study by Katafuchi et al. (2018) collected data from patients who had implants placed in a university setting (Washington) during the period of 1998 to 2003. Yi et al. (2020) collected data from patients who had implant restorations placed between March 2002 and February 2012 at Seoul National University Dental Hospital. Inoue et al. (2020) described patients who received implant treatment in the molar region at the Department of Fixed Prosthodontics, Osaka University Dental Hospital, and who visited for maintenance from May 2013 to August 2018.

### 3.3 Study characteristics and heterogeneity

The number of included patients in the three selected studies varied from 96 to 169. Included implants ranged from 168 to 349 in the three studies. In all three studies, the EA was measured by drawing a line parallel to the long axis of the implant and a line tangent to the restorative platform. The intersecting angle was considered the EA. Katafuchi et al. (2018) and Yi et al. (2020) measured the EA radiographically from the mesiodistal aspect and assigned this as an EA above and/or equal to 30° or below and/or equal to 30°. EP were radiographically determined and classified as straight, concave, and convex. Prevalence of peri‐implantitis was measured at the patient level, implant level, and surface level (mesial and/or distal). Inoue et al. (2020) measured the EA in four directions (mesial, distal, buccal, and lingual) on each implant body. Measurements in the mesiodistal direction were made from dental radiographs. Measurements of the buccolingual direction were made by overlaying the data of the study model as obtained with an optical scanner and digital imaging and communications in medicine data obtained by cone‐beam computed tomography imaging.

Katafuchi et al. (2018) included patients from 34 to 86 years with a mean age of 67.6 years. Implant types were grouped based on the tissue level and bone level, before analyzing EA and EP (Table 4). Peri‐implantitis was determined by bleeding on probing (BOP) and/or purulence, ≥2 mm of measurable bone loss after remodeling, and a pocket depth of ≥4 mm (Sanz & Chapple, 2012). Bone loss of 2 mm from the estimated marginal bone level after remodeling was chosen as a threshold. Measurement of the EA was done by blinding the marginal bone level from the examiner. EA measurement was repeated twice. From the bone‐level group, 76 implant restorations had a convex EP and 126 were classified as concave or straight. Of the tissue‐level implants, 50 restorations had a convex profile and 84 had a concave or straight profile. The EA of 48 the restorations on the bone level was >30° based on measurement outcome of one or more surfaces (mesial and/or distal) while 53 were ≤30°. The mean EA in the bone‐level, convex profile group was 37.5° with an SD of 11.7. Generalized estimating equation (GEE) models were used for the statistical analysis.

**Table 4:** Details distribution based on design aspect per implant group based on the study by Katafuchi et al. (2018)

Design aspect	Bone level	Tissue level
EA > 30°	48 implants (47.5%)	39 implants (58.2%)
EA ≤ 30°	53 implants (53.5%)	28 implants (41.8%)
Convex emergence profile	52 implants (51.5%)	34 implants (50.7%)
	76 surfaces	40 surfaces
Concave and straight emergence profile	49 implants (49.5%)	33 implants (49.3%)
	126 surfaces	84 surfaces

Abbreviation: EA, emergence angle.

In Yi et al. (2020), the age range was unknown, though the mean age was reported to be 59.8 years. The bone‐level implant group also distinguished internal from external abutment interface connections, subsequently creating three groups, as shown in Table 5, before analyzing the EA and EP. Peri‐implantitis was determined by comparing the current status with the baseline examination. The requirements to determine peri‐implantitis were, increased BOP and/or purulence, increased pocket depth, and at least 0.5 mm bone loss from the marginal bone level (Caton et al., 2018). Patients were only included if standardized X‐rays were available, meaning both initial and follow‐up radiographs as taken by using the paralleling technique. The blinding of the examiner during EA measurement was not described. EA measurements were repeated three times. The mean EA was estimated to be 27.9 with an SD of 14.2° for all implant groups. The most frequent EP was convex in bone‐level and tissue‐level implants combined (46.7%, *N* = 326 surfaces), followed by straight (32.2%, *N* = 225 surfaces) and concave (21.1%, *N* = 147 surfaces). In total, 298 implant restoration surfaces were classified as EA ≥ 30° and 400 restoration surfaces as EA < 30° (tissue‐level and bone‐level groups combined). Multivariable GEE logistic regression models were used for the statistical analysis.

**Table 5:** Details distribution based on design aspect per implant group based on the study by Yi et al. (2020)

Design aspect	Bone‐level internal	Bone‐level external	Tissue level
EA ≥ 30° (*N* = 298)	97 surfaces	177 surfaces	23 surfaces
EA < 30° (*N* = 400)	127 surfaces	253 surfaces	21 surfaces
Convex emergence profile (*N* = 326)	96 surfaces	201 surfaces	29 surfaces
Concave and straight emergence profile (*N* = 372)	128 surfaces	229 surfaces	15 surfaces

Abbreviation: EA, emergence angle.

In Inoue et al. (2020), the mean age was reported to be 61.9 ± 11.9 years. Marginal bone loss as a parameter for the presence of peri‐implantitis was measured around the implant from dental radiographs. The blinding of the examiner during EA measurement was not described. The measurement for the EA was performed once for each implant body by two measurers, and the interrater reliability was over 0.9, indicating a high level of reliability. The average EA from the buccolingual aspect was between 29.0° and 29.9° and from the mesiodistal aspect between 26.7° and 26.8°.

### 3.4 Quality assessment

Quality assessment is detailed in Supporting Information: Appendix S2. A study could be scored with a maximum of one star for each numbered item within the selection and exposure categories. A maximum of two stars could be given for comparability (Wells et al., 2019). Studies with the highest quality could score up to 10 stars. Katafuchi et al. (2018) were awarded 6/10 stars, Yi et al. (2020) with 5/10 stars, and Inoue et al. (2020) with 3/10 stars. The combined average value of the quality is 4.6. The quality of the studies is described in Table 6.

**Table 6:** Modified quality assessment of case‐control studies according to the Newcastle–Ottawa scale

Quality assessment criteria	Explanation of criteria	Katafuchi et al. (2018)	Yi et al. (2020)	Inoue et al. (2020)
*Selection*				
Is the case definition adequate?	With independent validation (radiographical and/or clinical measurement and determination of emergence angle (EA) and peri‐implantitis)	★	★	★
Representativeness of the cases	All cases with outcome of interest (peri‐implantitis)	★	★	**–**
Selection of controls?	Community controls	**–**	**–**	**–**
Definition of controls	Clear definition and no peri‐implantitis	**–**	**–**	**–**
*Comparability*				
Subjects in different outcome groups are comparable, based on study design or analysis. Confounding factors are controlled.	A) Study controls for EA and peri‐implant disease or inflammation of peri‐implant tissue B) Study controls for any additional factor	★	★	★
*Exposure/outcome*				
Ascertainment of exposure?	A) Professional diagnosis of peri‐implantitis or inflammation of peri‐implant tissue and adequate assessment of the EA B) Evaluators were blinded/did not know peri‐implant health status during EA measurement	★★	★	★
The same method of ascertainment of cases/controls?	The statistical test is used to analyze the data for all groups	★	★	–
Nonresponse rate	Same for all groups	–	–	–
Score		**6**	**5**	**3**

### 3.5 Synthesis of the results

#### 3.5.1 Prevalence of peri‐implantitis

An overview of study outcomes, peri‐implantitis prevalence based on implant groups, and implant aspects can be found in Table 3. The mean prevalence of peri‐implantitis at the implant level was 7.5% for tissue‐level implants and 22.8% for bone‐level implants in the study by Katafuchi et al. (2018) with an overall prevalence of 16.7%. In the study by Yi et al. (2020), the mean prevalence was 24.8% based on all implant groups combined. Separated by one‐ and two‐stage protocols, this was 21.2% and 29.1%, respectively.

Inoue et al. (2020) describe the relation of EA to peri‐implant tissue and assesses this based on BOP scores and marginal bone loss. However, no data on these parameters were provided and but *p*‐values of odds were given.

#### 3.5.2 Emergence angle

Two studies showed a higher prevalence of peri‐implantitis if the value of the EA was >30° compared to an EA < 30° (Katafuchi et al., 2018; Yi et al., 2020). Inoue et al. (2020) found that marginal bone loss tended to be smaller when the EA was around 20°–40°, although this difference was not significant (*p* = .06) (Table 3).

In the study by Katafuchi et al. (2018), the prevalence of peri‐implantitis for bone‐level implants with an EA > 30° was 31.3%, and 15.1% for bone‐level implants with an EA ≤ 30°. In the study by Yi et al. (2020), the highest peri‐implantitis prevalence based on implant surfaces was found in the bone‐level external connection group with an EA ≥ 30 (55.9%). For implants with an EA < 30° in the bone‐level external connection group, the prevalence was 11.5% (implant surface‐level). Both of these studies found no significant association between EA and EP in relation to peri‐implantitis for tissue‐level implants.

#### 3.5.3 Emergence profile

Inoue et al. (2020) did not evaluate EP. Katafuchi et al. (2018) showed a higher percentage (28.8%) of peri‐implantitis prevalence in convex profile types compared to other profile types (Table 3), the risk of peri‐implantitis in association with a convex profile as an isolated factor was not significant (*p* = .12). However, EP and EA interacted significantly with each other (*p* = .003). Therefore, a convex EP was found to be an additional risk for peri‐implantitis. A convex profile combined with an EA > 30° led to a peri‐implantitis prevalence of 37.8%. Yi et al. (2020) found a significantly higher prevalence of peri‐implantitis in association with convex restorations compared to other restoration profiles (*p* < .05) They observed a 46.6% prevalence of peri‐implantitis if EA ≥ 30 was combined with a convex profile compared to an 8.9% prevalence with convex EP and an EA < 30. No significant association between EP and tissue‐level implants was found in both of these studies.

### 3.6 Data analysis

The outcomes of the included studies could not be combined in a meta‐analysis because they were not homogenous. Also, mean data with standard deviations of peri‐implantitis prevalence were not provided. Therefore, heterogeneity could also not be statistically analyzed and was, therefore, only based on methodological and clinical variables. Data synthesis was mainly descriptive.

In an attempt to summarize the data, the weighted mean of the study results was calculated for which the underlying data can be found in Table 3. As the prevalence was measured at the implant level in Katafuchi et al. (2018) and at the surface level in Yi et al. (2020), the weighted mean prevalence of peri‐implantitis in relation to the EA was determined separately for each study. Calculation of the weighted mean in relation to the EP could combine data from both these studies because the prevalence of peri‐implantitis was determined at the implant level. The weighted mean percentage of peri‐implantitis for the convex EP was 35% and for the concave and straight EP, this was 12.5%.

### 3.7 Grading the body of evidence

Aspects used to rate the quality of evidence according to GRADE are shown in Table 7 and further detailed in Supporting Information: Appendix S3. Data from the included studies were consistent. The precision of data was considered “imprecise,” because one study, in particular, had an uneven distribution of implant design‐related aspects in the population. Outcomes could have been overestimated for EP, for example, because convex EP was the most frequent profile type. Therefore, data may not have been fully precise. The risk of bias was estimated moderate to high. Confounding factors were not fully excluded and may have influenced results and possibly led to a risk of bias. Generalizability was considered to be restricted because data from all three studies were derived from a university setting. The presence of reporting bias could not be analyzed because only three studies were found eligible but can also not be ruled out. Taking all GRADE aspects as presented in Table 7 into account relative to a moderate to the large magnitude of difference in weighted mean prevalence, the certainty of the results emerging from this review was estimated to be “very weak to weak.”

**Table 7:** Certainty assessment according to GRADE guidelines for peri‐implantitis in relation to a larger emergence angle (≥30°) and a convex emergence profile

Aspects of determining certainty	Outcome
The number included studies	3 (Figure 1)
Study design	Case‐control/cross‐sectional (observational) (Table 3)
Risk of bias	Moderate to high (Supporting Information: Appendix S3)
Precision of data	Imprecise
Consistency of results	Mostly consistent
Directness of evidence	Restricted generalizability
Reporting bias	Possible
The magnitude of the risk	Moderate to large (weighted mean)
Certainty	Weak (based on the above)
The direction of the evidence	There is “weak” evidence that an abutment emergence angle >30° is associated with a higher risk of peri‐implantitis or marginal bone loss. There is very weak evidence for a relationship between a convex emergence profile and a higher risk of peri‐implantitis.

Abbreviation: GRADE, grading of recommendations, assessment, development, and evaluation.

## 4 DISCUSSION

### 4.1 Answer to the focused question

Peri‐implantitis is a complex disease. Factors inducing peri‐implantitis can, for example, be plaque‐related and/or prosthetically related (Canullo et al., 2016). The aim of this systematic review was to summarize the scientific literature concerning the effect of the implant‐abutment EA and EP on the prevalence of the peri‐implant disease. Within the limitations of the available literature, there seems to be a significant relationship between a larger EA (>30°) and a higher prevalence of peri‐implantitis compared to a smaller EA (<30°) in bone‐level implants according to the included studies (Inoue et al., 2020; Katafuchi et al., 2018; Yi et al., 2020) and according to the findings by Inoue et al. (2020), an EA around 20° is probably less ideal. However, only three eligible studies were found that addressed EA in relation to peri‐implant health. A large EA may lead to overcontouring of the restoration design. The available scientific literature describes the influence of restoration contour on plaque accumulation and oral hygiene measures, suggesting that overcontoured restorations may lead to hindering hygienic efforts and more plaque accumulation. In teeth, overcontouring can affect the severity of periodontitis. However, there is a lack of information on the specific relation between overcontouring and peri‐implant disease (Dixon & London, 2019; Katafuchi et al., 2018; Koutouzis, 2019; Yi et al., 2020). It is a prosthetic factor that is yet, not highlighted enough, considering the plausible relation with peri‐implantitis. Literature also suggests that the EA and EP can be affected by platform switching design, implant placement, and implant position (Dixon & London, 2019; Katafuchi et al., 2018; Koutouzis, 2019; Yi et al., 2020). Dental care professionals and dental technicians can benefit from understanding the possible relation between EA and peri‐implantitis, and the factors that can influence the EA and restoration profile.

Studies show that the EA can be larger in platform switched implant designs compared to those that are not (Katafuchi et al., 2018). Theoretically, a platform switch implant, where the abutment diameter is smaller than the implant diameter, is assumed to reduce peri‐implant bone loss during remodeling (Dixon & London, 2019; Scutellà et al., 2015). Bone loss reduction in platform switching implants is caused by a greater horizontal distance between the alveolar bone and the implant abutment and is, therefore, farther away from bone compared to implants with matching abutment platform designs. Therefore, less bone remodeling is needed for the platform switched implant. However, the undersized platform may lead to an excessively contoured restorative profile, creating difficulty for probing measurements. There may be a possibility for higher risk of peri‐implantitis to occur if the platform switched implant design leads to a large overly contoured restauration (Dixon & London, 2019). Katafuchi et al. (2018) differentiated between supracrestal, crestal, and subcrestal placement of bone level implants and found the mean EA to be 28.9°, 29.0°, and 22.3°, respectively.

However, they found no association between implant depth and prevalence of peri‐implantitis.

Implant placement and position, for example, implant depth, can also be of influence the EA of the abutment restoration. The literature suggests that both convexities of the cervical restorative contour and EA are influenced by the buccopalatal position of the implant. The more palatal the implant is placed, the greater the EA and cervical contour. Placing an implant more buccally can be beneficial to creating a natural contour and EA (Scutellà et al., 2015). If an implant is placed more vertically (shallow), the angle of EA may need to be larger to achieve an adequate prosthetic design (Dixon & London, 2019). An implant platform should be placed at least 3 mm deeper than the cementoenamel junction of the future prosthetic design for normal development of the biologic width and EP (Schneider et al., 2018). However, placement of only 3 mm in depth is not always applicable. The narrower the implant platform, the more likely the need for an implant to be placed deeper in a vertical position to ensure a natural EP and a decreased restoration angle (Dixon & London, 2019). There is no direct proof that implant placement depth is associated with peri‐implantitis (Katafuchi et al., 2018). However, understanding the relation of implant placement and the possible effect on the EA can be of the essence to the restorative design and may be indirectly related to improving peri‐implant health.

Interestingly, a recent retrospective study evaluated the influence of restorative design on the progression of peri‐implant bone loss (Majzoub et al., 2021). Included patients had at least one functionally loaded, single bone level implant diagnosed with peri‐implantitis. The selection also included no evidence of peri‐implantitis documented during a maintenance visit ≤6 months before its diagnosis. Data analysis showed positive relationships between marginal bone loss and restoration EA. Implants with a restoration EA of >30° had 2.33 ± 1.20 mm marginal bone loss between the diagnosis of peri‐implantitis and the previous assessment ≤6 months earlier, whereas implants with a restoration EA of ≤30° had 0.59 ± 0.71 mm marginal bone loss (*p* < .001).

In the included studies, tissue‐level implants showed a lower prevalence of peri‐implantitis and no significant peri‐implant disease association in relation to EA and EP (Katafuchi et al., 2018; Yi et al., 2020). A possible reason could be that the implant platform is at the tissue level and not below such as in bone‐level implants. Therefore, a larger EA and convex EP may not be of impact on the peri‐implant tissue (Katafuchi et al., 2018). Another reason could be that bone loss can be related to where the implant‐abutment interface is located. In comparison with bone‐level implants, tissue‐level implants do not have an interface at the bone crest (Sasada & Cochran, 2017). Therefore, initial contamination of bacteria does not occur at the bone crest in tissue‐level implants as there is no microgap at the marginal bone level (Sasada & Cochran, 2017). Within the microgap, which is the space between the implant‐abutment connection and the implant itself, bacteria can colonize and form a plaque layer. This can subsequently cause inflammation that can ultimately lead to bone loss (Tallarico et al., 2017).

### 4.2 Limitations and strengths

Only three studies were found eligible. Also, high‐quality study designs, like RCT's were not found.

Generalizability is questionable, publication bias cannot be ruled out and the risk of bias may be present.

The convex EP, as an isolated factor, showed a larger association with peri‐implantitis in the study by Yi et al. (2020) but was also the most frequent EP type to occur in Yi et al. (2020). Selection bias may occur if case and control groups are not representative of the general population (Lopez et al., 2007). Study groups may have had a higher prevalence of peri‐implantitis than the general population because they were selected based on the outcome of interest. Inoue et al. (2020) did not provide data concerning marginal bone level nor criteria with respect to peri‐implantitis. Yi et al. (2020) determined the presence of peri‐implantitis according to the new classification of the European Federation of Periodontics (EFP) by Caton et al. (2018). Katafuchi et al. (2018) defined peri‐implantitis based on an older classification of the EFP (Sanz & Chapple, 2012), which was the most recent classification at the time the research was conducted. The diagnosis of peri‐implantitis may not have been similar for both of these studies. The thresholds for pocket depth and bone loss in the study by Katafuchi et al. (2018) were lower than the thresholds described in the recent EFP classification. False‐positive outcomes or inconsistency in peri‐implantitis diagnosis cannot be ruled out. As peri‐implantitis is a multifactorial disease all other etiological factors and risk factors are not fully excluded in the results of both studies. A causal relationship between EA, EP, and peri‐implantitis cannot easily be made. Confounding variables, for example, plaque and oral hygiene, may have affected the outcome. One study (Katafuchi et al., 2018) did not report on oral hygiene and plaque control of the included patients.

One of the exclusion criteria for this review was (partial) dentures. Namely, a study on risk indicators for peri‐implantitis (Dalago et al., 2017) documented a higher risk in implants supporting complete dentures than in those supporting single crowns. More recently, a retrospective study after 17–23 years (Kesar et al., 2021) also found peri‐implantitis and increased bone loss more frequently in implants supporting removable prostheses.

### 4.3 Implications for future research

The study results suggest a correlation between a larger abutment EA and peri‐implantitis. To further clarify this relationship, more high‐quality evidence research is needed. A report (Schwarz et al., 2021) and review article (Mattheos et al., 2021) also discuss the relation between EA and EP in relation to peri‐implantitis. Both papers carefully suggest the possibility of a higher peri‐implantitis prevalence due to a larger EA in combination with a convex EP, also based on the papers of Katafuchi et al. (2018) and Yi et al. (2020). Studies should not only be performed on a long‐term basis, but also on a larger spectrum with patients included from multiple settings. For further research, it is advisable that groups should be based on even distribution of implant types and better isolation of factors. Furthermore, the effect of platform switching on the EA should also be included in future research, considering the possible clinical relevance on the restoration profile and the association with peri‐implant health.

## 5 CONCLUSION

Limited research is available on the EA of the implant‐abutment in relation to peri‐implant disease. Only three eligible studies were found. Reported associations based on this review should therefore be considered with caution. Based on the results of this review, there seems to be an association between a larger EA (>30°) and a higher prevalence of peri‐implantitis or marginal bone loss compared to a smaller EA (<30°).

A convex EP may also be associated with a higher prevalence of peri‐implantitis. However, for both these prosthetic design aspects, the research is limited and causality remains a question. For future research, long‐term studies with a larger sample size are necessary.

## AUTHOR CONTRIBUTIONS

Sara Soulami contributed to the design, search, and selection, and drafted the manuscript. Dagmar E. Slot contributed to the analysis and interpretation and critically revised the manuscript. Fridus van der Weijden contributed to conception and design, search and selection, analysis and interpretation, and critically revised the manuscript. All authors gave final approval and agreed to be accountable for all aspects of the work ensuring integrity and accuracy.

## CONFLICT OF INTEREST

The authors declare no conflict of interest.

## Supporting information

Supporting information.

Supporting information.

Supporting information.

## Data Availability

Data analyzed in this study were a reanalysis of existing data, which are openly available at locations cited in the reference section.
