# Supplementary material for: Implant‐abutment emergence angle and profile in relation to peri‐implantitis: A systematic review
Source: Clin Exp Dent Res. 2022 Jun 17;8(4):795–806. doi: 10.1002/cre2.594 (PMC9382038; doi:10.1002/cre2.594)

**Appendix S1. Ethical approval by the ethical comission board of the Academic Centre of Dentistry Amsterdam (ACTA).**


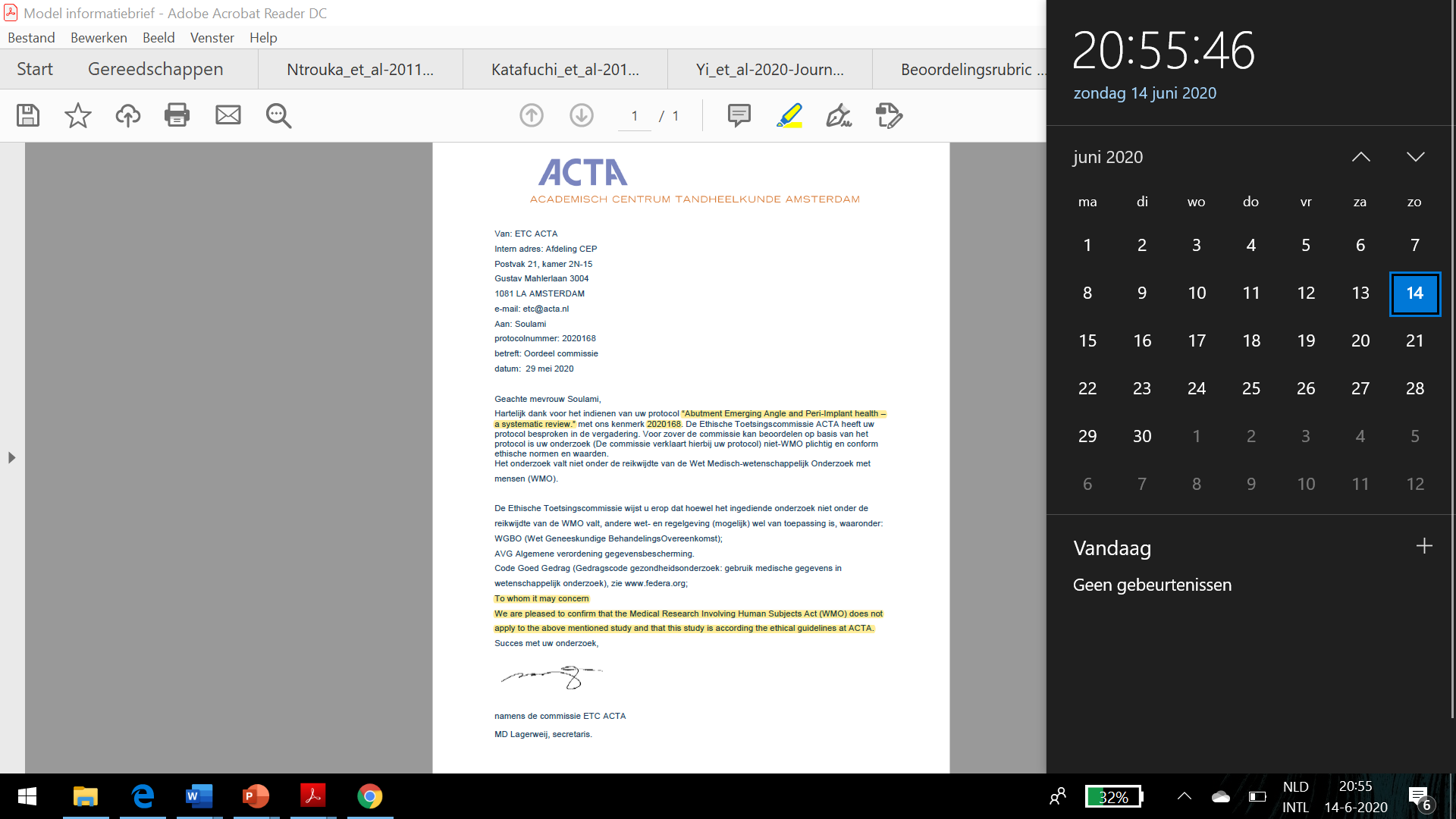

Supplement: Supplementary file 1 — Supporting information. [file CRE2-8-795-s001.docx]
