# Supplementary material for: Implant‐abutment emergence angle and profile in relation to peri‐implantitis: A systematic review
Source: Clin Exp Dent Res. 2022 Jun 17;8(4):795–806. doi: 10.1002/cre2.594 (PMC9382038; doi:10.1002/cre2.594)
Supplement: Supplementary file 2 — Supporting information. [file CRE2-8-795-s003.docx]

**Appendix S1. Modified method of assessing the quality of case control studies based on the New Castle Ottawa Scale (NOS).**

**Selection assessment**

1. Is the case definition adequate?
2. Yes, with independent validation (radiographic analysis and clinical assessment of emergence angle and peri-implantitis) ★
3. Yes, e.g. record linkage or based on self-reports
4. No description
5. Representativeness of the cases
6. Consecutive or obviously representative series of cases ★
7. Potential for selection biases not stated
8. Selection of controls
9. Community controls ★
10. Hospital or (dental) university clinic controls
11. No description
12. Definition of controls
13. No history of disease (peri-implantitis) ★
14. No description of source

**Comparability assessment**

1. Comparability of cases and controls based on the design or analysis
2. Study controls for emerging angle and peri-implantitis ★
3. Study controls for any additional factor ★

**Exposure assessment** 1) Ascertainment of exposure

1. Secure record (e.g. patient records) ★
2. Evaluators are blinded during measurement of emergence angle (did not no status of peri-implant disease) ★
3. Self-report or medical record only
4. No description
5. Same method of ascertainment for cases and controls
6. Yes ★
7. No
8. Non-response rate
9. Same rate for all groups ★
10. Non responders described
11. Rate different and no designation
