# Supplementary material for: Implant‐abutment emergence angle and profile in relation to peri‐implantitis: A systematic review
Source: Clin Exp Dent Res. 2022 Jun 17;8(4):795–806. doi: 10.1002/cre2.594 (PMC9382038; doi:10.1002/cre2.594)
Supplement: Supplementary file 3 — Supporting information. [file CRE2-8-795-s002.docx]

**Appendix S3. Quality of evidence grades, factors that determine, reduce, and increase the quality of evidence.**

| **Table 5.1: Quality of Evidence Grades** | |
| --- | --- |
| Grade | Definition |
| High | We are very confident that the true effect lies close to that of the estimate of the effect. |
| Moderate | We are moderately confident in the effect estimate: The true effect is likely to be close to the estimate of the effect, but there is a possibility that it is substantially different |
| Low | Our confidence in the effect estimate is limited: The true effect may be substantially different from the estimate of the effect. |
| Very Low | We have very little confidence in the effect estimate: The true effect is likely to be substantially different from the estimate of effect |

| **Table 5.2: Factors that can reduce the quality of the evidence** | |
| --- | --- |
| Factor | Consequence |
| Limitations in study design or execution (risk of bias) | **↓**1 or 2 levels |
| Inconsistency of results | **↓**1 or 2 levels |
| Indirectness of evidence | **↓**1 or 2 levels |
| Imprecision | **↓**1 or 2 levels |
| Publication bias | **↓**1 or 2 levels |

| **Table 5.3: Factors that can increase the quality of the evidence** | |
| --- | --- |
| Factor | Consequence |
| Large magnitude of effect | **↑** 1 or 2 levels |
| All plausible confounding would reduce the demonstrated effect or increase the effect if no effect was observed | **↑** 1 level |
| Dose-response gradient | **↑** 1 level |
